# Imaging Modalities for Evaluating Lymphedema

**DOI:** 10.3390/medicina59112016

**Published:** 2023-11-16

**Authors:** Bendeguz Istvan Nagy, Balazs Mohos, Chieh-Han John Tzou

**Affiliations:** 1 Department of Thoracic, Cardiac and Vascular Surgery, Westpfalz-Klinikum GmbH, 67655 Kaiserslautern, Germany; 2 Heart and Vascular Center, Semmelweis University, 1094 Budapest, Hungary; 3 Plastic and Reconstructive Surgery, Department of Surgery, County Hospital Veszprem, 8200 Veszprem, Hungary; 4 Balaton Private Clinic, 8200 Veszprem, Hungary; 5 Plastic and Reconstructive Surgery, Department of Surgery, Hospital of Divine Savior, 1060 Vienna, Austria; 6 Faculty of Medicine, Sigmund Freud University, 1020 Vienna, Austria; 7 Lymphedema Center Vienna, TZOU MEDICAL., 1060 Vienna, Austria

**Keywords:** lymphedema, lympho-venous anastomosis (LVA), lymphoscintigraphy (LS), near-infrared fluorescent (NIRF) imaging, indocyanine green (ICG), ultrasonography (US), magnetic resonance lymphangiography (MRL), computed tomography (CT), photoacoustic imaging (PAI), optical coherence tomography (OCT)

## Abstract

Lymphedema is a progressive condition. Its therapy aims to reduce edema, prevent its progression, and provide psychosocial aid. Nonsurgical treatment in advanced stages is mostly insufficient. Therefore—in many cases—surgical procedures, such as to restore lymph flow or excise lymphedema tissues, are the only ways to improve patients’ quality of life. *Imaging modalities:* Lymphoscintigraphy (LS), near-infrared fluorescent (NIRF) imaging—also termed indocyanine green (ICG) lymphography (ICG-L)—ultrasonography (US), magnetic resonance lymphangiography (MRL), computed tomography (CT), photoacoustic imaging (PAI), and optical coherence tomography (OCT) are standardized techniques, which can be utilized in lymphedema diagnosis, staging, treatment, and follow-up. *Conclusions:* The combined use of these imaging modalities and self-assessment questionnaires deliver objective parameters for choosing the most suitable surgical therapy and achieving the best possible postoperative outcome.

## 1. Introduction

Lymphedema is a progressive condition with protein-rich interstitial fluid accumulation due to impaired lymphatic drainage [1,2,3,4,5,6].

Its symptoms vary on a scale well presented in the International Society of Lymphology (ISL) staging system [7] (Appendix A). Recent studies have indicated that ISL staging, based solely on clinical signs, cannot describe lymphedema severity precisely, and it does not correlate well with quality of life (QoL) [7]. Therefore, the combination of various lymphedema evaluations provides a more accurate diagnosis, and imaging modalities are of the utmost importance.

Lymphedema assessment can be carried out using the following:

1. Objective measurements [5]: limb volume measurement by perometry/CT/water dis-placement, skin tonometry, bioimpedance spectroscopy (BIS, L-dex score), bioimpedance analysis (BIA), and tissue dielectric constant (TDC);
2. Imaging modalities (see Section 2);
3. Self-assessment questionnaires: lymphedema life impact scale (LLIS) [8], lower limb functional index (LLFI) [9], lower extremity functional scale (LEFS) [10], disabilities of the arm, shoulder, and hand (DASH) [11], International Classification of Functioning, Disability and Health (ICF) [12], and general quality of life (QoL) [5,7,13,14].

With more precise and earlier diagnosis, individualized lymphedema treatment will be able to efficiently reduce edema volume, prevent further fluid accumulation, and provide psychosocial support.

The gold standard treatment is complex physical therapy, also called complete decongestive therapy/CDT (manual lymphatic drainage, exercise, use of non-elastic wrappings and compressive garments, skin care) [2,4], intermittent pneumatic compression [2,15], compressive garments [16], heat therapy [2,4,17,18], and extracorporeal shock-wave treatment [19]. Pharmacological agents supporting lymph drainage are studied; currently, there is no specific, FDA/EMA-approved drug [6,18]. If any of the conservative therapies fail, an exact lymphedema evaluation will provide more information to design an individualized surgical treatment. In this regard, imaging modalities are crucial for diagnosis, staging, intraoperative imaging, and follow-up. Surgical therapy consists of conventional and microsurgical procedures, and it aims to increase lymph outflow of the swollen areas and—in the severest cases—to debulk the amount of excess tissue [5]. Lymph drainage can be enhanced (1) by anastomoses between the lymphatic and venous systems via direct lympho-venous anastomoses (LVA) or lymph-node-venous anastomoses (LNVA) [20]—in multiple centers as a preventative measure (LYMPHA); (2) by restoring lymph vessel patency in locally interrupted lymph systems with lympho-lymphatic anastomoses (LLA) or a venous segment interposition (LVLA) [21]; or (3) by vascularized lymph node (VLNT), vessel (VLVT) or system (LYST) transfer. Liposuction—possibly lymph vessel sparing— and surgical resection (“debulking”) aim to decrease the amount of excess fat, fibrotic subcutaneous tissue, and skin.

In this paper, the authors describe all current imaging modalities for diagnosis, therapy planning, and follow-up documentation and provide information on each of their indications for their usage, functionality, practicability, and limitations.

## 2. Imaging Modalities

### 2.1. Ultrasonography (US)

**Figure 1:**
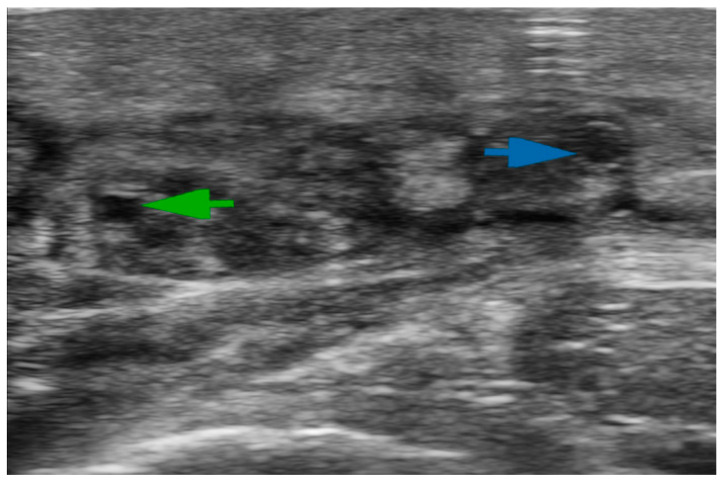
Ultra-high-frequency ultrasound (48 MHz probe) accurately reveals lymphatic vessels and neighboring veins. The green arrow indicates the lymphatic collector; the blue arrow shows the vein.

Ultrasonography (US) is the modality of choice to exclude venous components in lymphedema. Furthermore, it is a valuable tool for assessing lymphedema and tissue composition, identifying veins and lymphatic vessels suitable for LVA (Figure 1), and can also be useful for lymphatic tissue transfer by providing information on flap anatomy [22]. The latter examinations are frequently performed using high-frequency (HFUS) and ultra-high-frequency (UHFUS) (48–70 MHz) probes.

Increased dermal thickness and tissue stiffness due to fibrosis are characteristic of chronic lymphedema and can be examined via conventional B-mode US and ultrasound elastography [23], respectively. Sonographic localization of fluid and solid predominant regions in the lymphedematous limb is crucial since we expect the most volume reduction by positioning physiologic procedures (such as LVA surgeries and lymphatic tissue transfers) in the regions of excessive fluid accumulation. Preoperatively identifying adjacent superficial comparable-diameter venules [24] and ectasis-type lymph vessels—according to normal, ectasis, contraction, sclerosis type (NECST) classification [25]—significantly increases the success rate of LVA operations [24,25,26,27].

Contrast-enhanced ultrasonography (CEUS) is the most recent sonographic tool for detecting sentinel lymph nodes and lymphatic vessels [28,29,30]. CEUS, HFUS, and UHFUS are effective means of planning LVA procedures by revealing candidate venules, the most optimal regions, and lymphatic vessels, and are—in many cases—superior alternatives to ICG lymphography [28,31,32,33].

The limitations of the US are a steep learning curve, dependency on the examiner’s skill, and the low tissue penetration of UHFUS (23.5 mm for 48 MHz and 10 mm for 70 MHz [34]).

### 2.2. Lymphoscintigraphy (LS)

**Figure 2:**
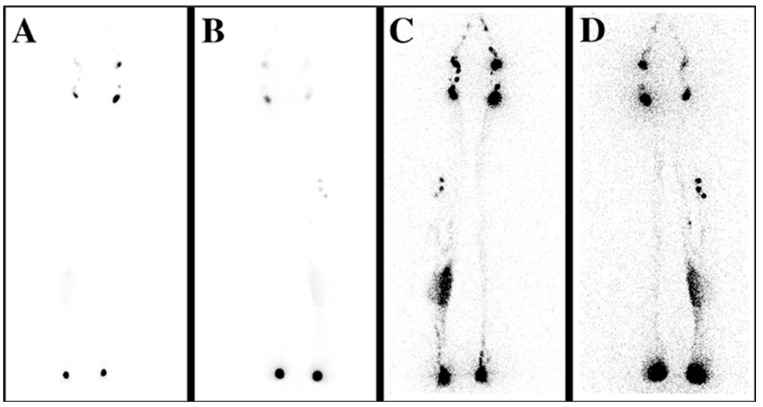
Lymphoscintigraphy (LS) reveals the lymphatic flow insufficiency. (**A**) Anteroposterior (AP) transmission LS scan shows the early distribution of the tracer; proximal lymph nodes can be observed. (**B**) Posteroanterior (PA) scan, early distribution. (**C**) AP LS scan demonstrates the late distribution of the isotope, showing lymphatic retention on the medial calf on the right side. (**D**) PA scan, late distribution.

Lymphoscintigraphy (LS) is a nuclear medicine modality, and it has been the gold standard for confirming the diagnosis of lymphedema for several decades. It is a procedure where a gamma-ray-emitting technetium 99 m labeled compound is injected intradermally/subcutaneously/subfascially, and its distribution is subsequently detected with a gamma camera. (Figure 2) Its limitations are the lack of standardized protocol, inferior spatial resolution, the presence of ionizing radiation, and accessibility. The lack of standardized protocol differences in the selection of the radiotracer, dose, injection site, acquisition time, and dynamic or static acquisition after rest or exercise deters the cross-center reproducibility of the lymphoscintigraphic results [35].

There are multiple 99 mTc-labeled tracers, such as albumin-nanocolloid, sulfur colloid, phytate, antimony sulfide, and so forth, with different particle sizes [35,36,37,38]. Furthermore, 50–70 nm is considered the optimal diameter since such particles can enter the lymphatic system but not the blood capillaries [38]. This value ranges between 10 and 100 nm, according to another publication [37].

Lymphoscintigraphy delivers both qualitative and quantitative interpretations. It describes the lymphatic morphology—recommended with colloidal tracers, such as the number of proximal lymph nodes, the quantity and course of lymphatic vessels, the presence of collateral lymphatic flow, and the characteristics of dermal backflow [35]. The measurement of the tracer uptake in the proximal lymph nodes, tracer clearance from the injection site, and appearance in the blood are the quantitative parameters of lymphoscintigraphy. Comparison between the affected and unaffected limb, if possible, provides invaluable data, both in qualitative and quantitative examinations. The relevance of quantitative lymphoscintigraphy is questionable in everyday use since the diagnosis of lymphedema can be confirmed via qualitative lymphoscintigraphy, the quantitative measurements are time-consuming, and their results are often inconsistent [39].

Greater sensitivity and better three-dimensional spatial resolution can be achieved by combining LS with single-photon emission computed tomography (LS-SPECT) or computed tomography [40].

**Figure 3:**
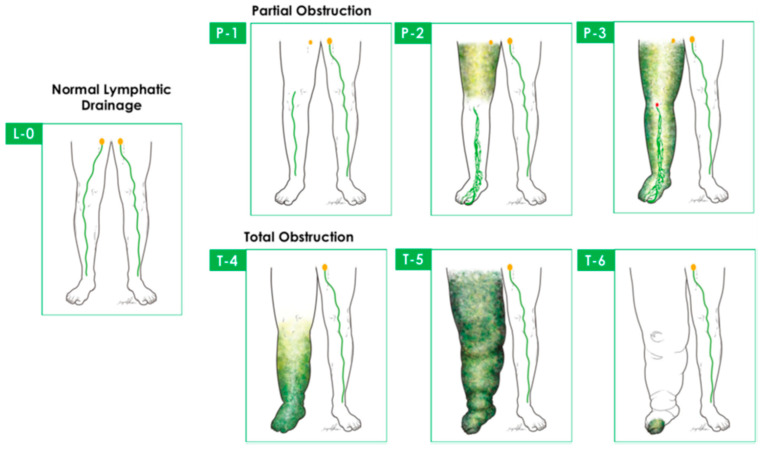
Taiwan Lymphoscintigraphy Staging differentiates lymphedema into 3 patterns and 7 stages: L0, normal drainage; P1–3, partial obstruction; T4–6, total obstruction. Cited with permission [41].

The available lymphoscintigraphic staging systems aiming to help decision making and follow-up are largely intricate for everyday interpretation, but they can be useful for specific indications. They do not have a clear relation to lymphedema severity and treatment outcome. The most recent LS staging system is the Taiwan Lymphoscintigraphy Staging (TLS), based on the visualization of (1) proximal/intermediate lymph nodes, (2) linear lymphatic vessels, and (3) dermal backflow; LS acquisition protocol is detailed in [40] (Figure 3). (TLS differentiates lymphedema into three patterns and seven stages: L0, normal drainage; P1-3, partial obstruction; T4-6, total obstruction.) Cheng’s Lymphedema Grading helps lymphedema surgical treatment-related decision making and is based on imaging (TLS, ICG findings) and clinical signs (duration of symptoms, circumferential and CT-based volumetry, and frequency of cellulitis) [41]; see Table 1.

### 2.3. Indocyanine Green Lymphography (ICG-L)

Indocyanine green (ICG) lymphography (ICG-L), also termed near-infrared fluorescent (NIRF) imaging, is a mainstay tool in many centers for diagnosing and assessing lymphedema. NIRF can detect superficial lymphatics (imaging depth: 1–2 cm) pre-, intra-, and postoperatively in real time. Therefore, it is a helpful tool for reconstructive surgery planning, intraoperative imaging, mapping, reverse mapping, and lymphatic vessel-sparing liposuction [5,42].

ICG is a fluorescent, water-soluble dye with a molecular weight of 774.96 Da and great affinity to plasma lipoproteins (maximum absorption wavelength: 790 nm; maximum emission wavelength: 835 nm). It has a low tissue and vascular wall permeability due to its binding to larger molecular weight plasma proteins (intensely to HDL, moderately to LDL) [43]. It was developed by Kodak Research Laboratories for examining cardiac output, but it gained relevance in assessing liver function, cerebral circulation, and retinal circulation. This sodium-iodine-containing chromodiagnostic solution is excreted in the liver and considered safe intravenously, and if extravasated, with a low incidence of complications; however, it should be used with caution in patients with liver and kidney disease and in dialyzed patients, and it should be omitted in patients with iodine sensitivity, thyroid tumor, or hyperactivity. It is FDA approved for intravenous use (in lymphography, ICG is used off-label) and was classified as pregnancy-risk category C; however, some studies contradict its toxicity for the fetus [44,45,46].

NIRF protocols vary in different centers. It is commonly administered in or around the lymphedematous area or in the second and fourth interdigital space of the extremities. The lymphatic uptake and flow can be promoted using the fill and flush technique [47] or active movement. Early distribution can be examined right after administration; the late plateau phase can be imaged 2 to 18 h after the injection. Patent, non-leaking lymphatic vessels are depicted as linear patterns, dilated lymphatics show as a splash, minor lymphatic extravasation is visualized as stardust, and extensive extravasation is expressed as a diffuse dermal backflow pattern.

A qualitative staging system was introduced based on the ICG dermal backflow patterns [48,49,50,51], as shown in Table 2 and Table 3. Though ICG staging does not correlate well with the ISL stage, bioimpedance spectroscopy findings, limb volume difference, and LLIS, it is a highly sensitive method to diagnose lymphatic dysfunction, even in a subclinical phase [14,51].

Quantifying ICG lymphography can be performed by measuring transit times [53] and contractility [54]. However, these quantitative ICG procedures have not gained widespread clinical acceptance.

**Figure 4:**
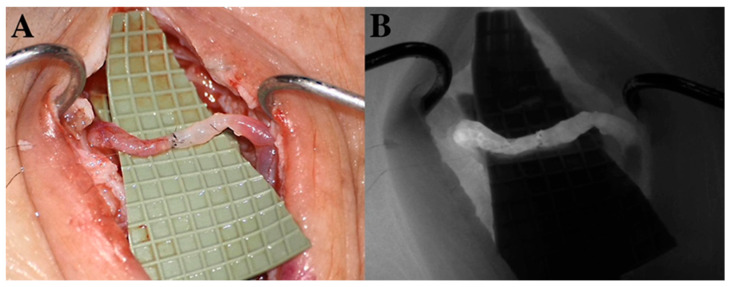
(**A**) Lymphovenous anastomosis (LVA). An ecstatic lymphatic vessel (left side) is anastomosed to a reflux-free vein (right side). Wash-out of blood can be observed from the distal part of the vein to the patent venous valve. (**B**) Microscope-integrated intraoperative ICG-L ensures the patency of lympho-venosus anastomosis.

**Figure 5:**
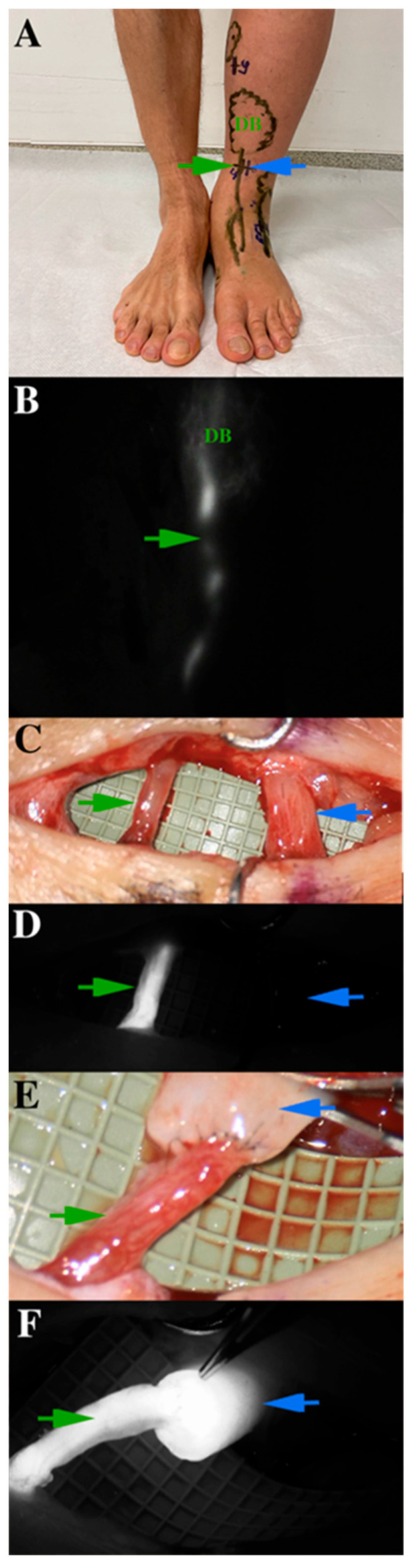
Presentation of indocyanine green (ICG) lymphography on a specific lymphatic vessel, which is for lymphovenous anastomosis operation. (**A**) ICG lymphography markings on the skin (the green arrow indicates the lymphatic vessel, the blue arrow shows at the vein). Linear and dermal backflow (DB) patterns are indicated differently. (**B**) ICG lymphography patterns on the same region. (**C**) The previously detected lymphatic collector is dissected and verified under microscope. (**D**) Microscope-integrated ICG lymphography shows the functional lymphatic vessel. (**E**) The anastomosis is made with 11-0 non-absorbable monofil sutures. (**F**) Microscope-integrated ICG lymphography ensures the patency of the anastomosis and excludes any leakage. Lymphatic flow washes out blood from the vein.

Indocyanine green lymphography (ICG-L) is useful for planning LVA procedures; microscope-integrated ICG-L can facilitate intraoperative dissection and ensure the patency of the anastomosis (Figure 4 and Figure 5).

The limitations of ICG imaging are its small detection depth, the suboptimal parameters of the ICG molecule (low quantum yield, poor stability, self-quenching), and its iodine content [37]. Furthermore, it can visualize just those parts that drain from the injection site.

### 2.4. Magnetic Resonance Imaging Lymphography (MRL)

**Figure 6:**
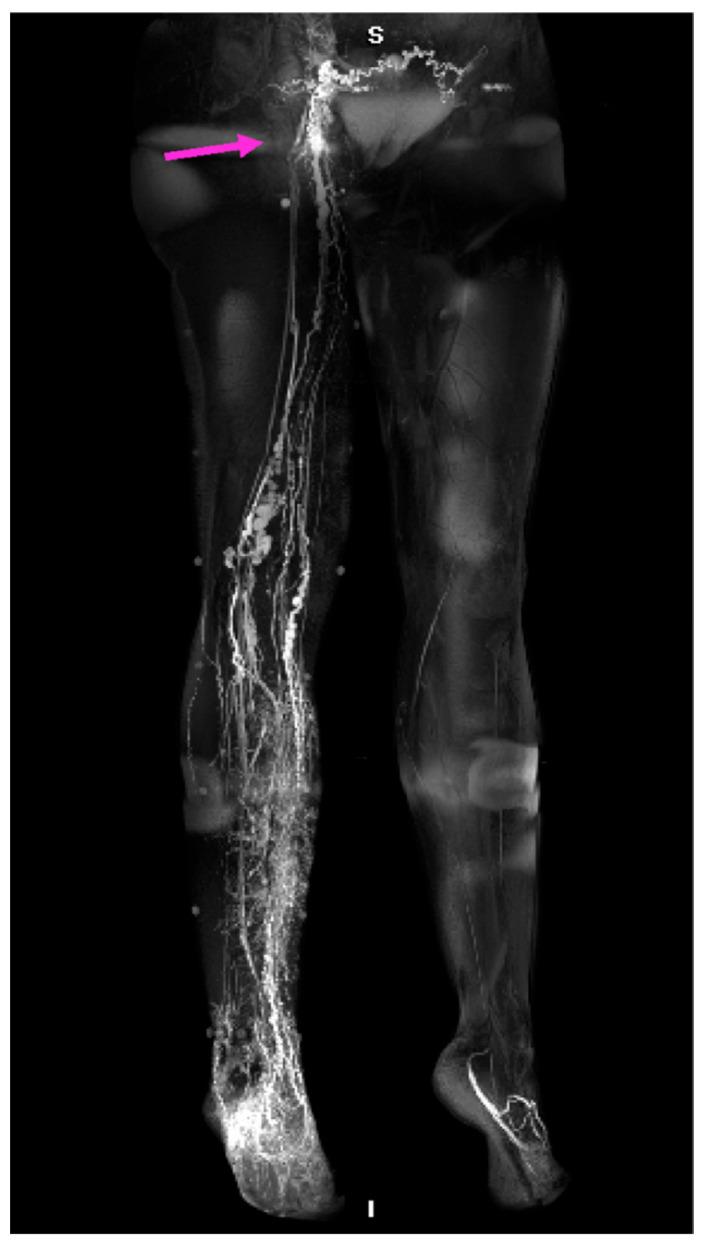
MRL of a patient with lower limb primary lymphedema. Hyperplasic lymphatic vessels and inguinal lymph nodes (arrow) of the right lower extremity are visible [55].

Magnetic resonance imaging lymphography (MRL) is a highly sensitive tool for diagnosing fluid accumulation and adipose hypertrophy. It reveals the location of lymphatics, depicts the extent of dermal backflows, and is useful in identifying venous obstruction and occult metastases in 3D without radiation exposure, as shown in Figure 6 [37,51,55,56].

Non-contrast MRI lymphography uses heavily T2-weighted sequences to highlight signals from the accumulated fluid and depress the signal from solid tissues. It has been utilized to visualize central lymphatics efficiently [57]. Chemical exchange saturation transfer (CEST) MRI uses the protons of amide groups in protein-rich interstitial environments as a contrast, thus being specific for protein-rich interstitial fluid accumulation in lymphedema [58]. For the further enhancement of lymphatic specificity of non-contrast MRI, arterial spin labeling was attempted to assess lymphatic flow velocity [37,56,59].

Contrast-enhanced magnetic resonance lymphography starts with T2-weighted pre-tracer imaging. Subsequently, a gadolinium-based tracer is injected interstitially, and the positive enhancement signal is detected on T1-weighted images. The contrast agent can be administered intranodal, as with X-ray lymphography, for a better visualization of the central or hepatic lymphatics [37]. The Gd-based MR contrast agents have a low molecular weight (<1 kDa). Therefore, they are absorbed by blood vessels and lymphatic vessels, posing difficulty in differentiating between these vascular systems on contrast-based MRL images. Differences between the anatomy of these systems, the delayed uptake in lymphatics compared to blood vasculature revealed on a series of dynamic images, or a second MRI with an intravenous administration of the tracer (delayed MR lymphogram) can help the identification. Furthermore, the so-called dark-blood technique helps differentiate lymphatics and blood vessels by applying an iron-based tracer intravenously and a Gd-based tracer intradermally. The iron-based contrast agent suppresses the venous enhancement [60].

MRL is a valuable tool in confirming the diagnosis of lymphedema and a supportive tool in lymphatic surgery planning [60]. It helps differentiate between fluid- or solid-predominant areas; therefore, it supports identifying the areas that may benefit from reconstructive surgery or excess-tissue removal. Furthermore, it is an invaluable tool in lymphatic vessel and vein mapping before LVA procedures and in donor- and recipient-site assessment in free lymphatic tissue transfer.

The limitations of MRL include its affordability, extensive exposure time, the risk of contrast allergy, and contraindicated utilization in patients with severe kidney disease or metal implants. Furthermore, the low specificity of the contrast agent for lymphatics due to the low molecular weight of the tracer makes selective MR lymphography difficult and emphasizes the need for improved lymphatic-specific tracers [37].

An MRL staging system was reported, but its clinical relevance is still under investigation [61].

### 2.5. Computed Tomography (CT)

Computed tomography and computed tomography angiography have a supportive role in lymphedema diagnostics. They can detect venous etiology and incidental malignancies and are useful in locating perforator vessels during the preparation for lymphatic tissue transfer [62]. CT can evaluate the excess fibrous tissue and the presence and severity of edema; however, it cannot differentiate between lymphedema and edema [56].

### 2.6. Photoacoustic Imaging (PAI)

Photoacoustic imaging or optoacoustic imaging—a promising imaging modality—combines optical absorption and ultrasound detection (“light in, sound out”). The examined object is illuminated with non-ionizing, short-pulsed light. The absorbed light generates heat and thermal expansion in the illuminated tissue, which in turn creates ultrasound waves. Therefore, this scalable method captures light-absorbing components (e.g., melanin, hemoglobin, ICG) in real time.

Photoacoustic microscopy provides high spatial resolution at the cost of detection depths, while photoacoustic tomography can penetrate several cm deep but provides imaging with lower resolution.

Knowledge of the absorption spectra of the molecules of interest and the background allows multispectral imaging—multispectral optoacoustic tomography (MSOT)—thus imaging the lymphatics and blood vessels simultaneously [37,63,64,65].

**Figure 7:**
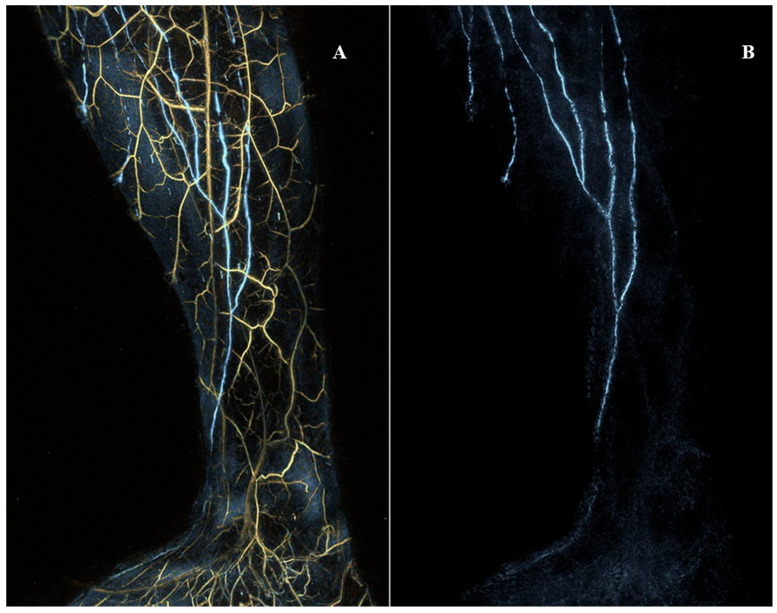
The medial view of the right lower extremity of a woman in her thirties without any past medical history registered with photoacoustic/optoacoustic lymphangiography. (**A**) Both venules and lymphatic vessels are shown. (**B**) Only lymphatic vessels are shown. Cited with permission. [65].

Kajita et al. visualized lymphatics 0.2 mm in diameter at a maximal depth of approximately 2 cm with a robust imaging appliance capable of detecting at two wavelengths [64,66], as shown in Figure 7. Giacalone et al. used a handheld device with similar tissue penetration parameters and capability to monitor seven wavelengths for observing the position and contractility of lymphatic vessels, assuring ICG mapping, and selecting incision sites before LVA [63].

PAI allows the exact visualization of lymphatic vessels without ionizing radiation exposure, in real time, even in the regions of dermal backflows, where the NIRF camera detects just splash or diffuse ICG accumulation.

Despite its limited penetration (approx. 2 cm), PAI is a promising tool and has the potential to gain widespread utilization in medicine.

### 2.7. Optical Coherence Tomography (OCT)

Optical coherence tomography (OCT), also called laser tomography, is an optical tomographic imaging modality based on low-coherence interferometry; basically, the backscattering is detected from an illuminated object (similarly to B-mode in ultrasound).

OCT was first introduced for ex vivo imaging in 1991 and applied in medicine later in that decade [67,68]. Since then, it has been a mainstay tool in ophthalmology for examining the tissues of the fundus and is becoming a standard tool for atherosclerosis assessment in interventional cardiology [68]. As a potential imaging modality, it was recently introduced in dermatology [69] and lymphology [70]. The probing depth reaches 2 cm in transparent scattering media (e.g., eye) and 1–2 mm in highly scattering tissues (e.g., skin) at a micrometer-precise resolution [67].

**Figure 8:**
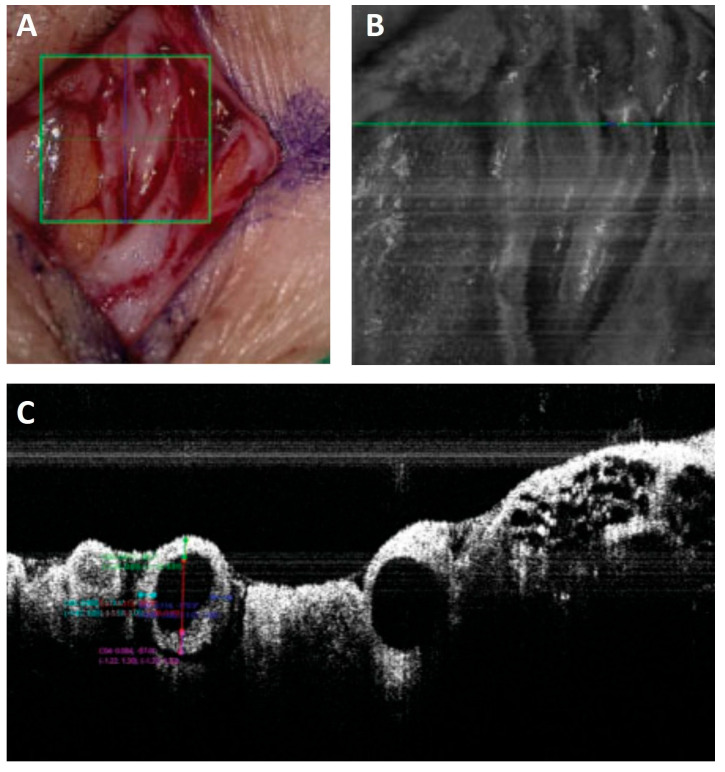
Lymphatic vessel photographed (**A**), the green square in A shows the area imaged with a microscope-integrated NIRF camera (**B**) and the green line in B indicates the position of the crossectional image captured with a microscope-integrated OCT (**C**). The diameter and wall-thickness of vessels can be measured precisely on the OCT images [70].

In the microscope-integrated OCT system utilized intraoperatively in LVA surgeries by Hayashi et al., this modality has an imaging depth of 2.5 mm and an axial resolution of less than 4 μm [70] and provides information on the number of lymphatic lumens in a dissected area, wall thickness, diameter, luminal obstruction, and valves of lymphatic vessels and improves the patency of LV anastomosis (Figure 8).

Currently, ICG-enhanced lymphatic vessels are considered functional and applicable for LVA. Yang et al. proposed that lymphatic-flow-positive but non-ICG-enhanced lymphatic vessels could be considered functional and suitable for LVA [71]. In this regard, OCT has the potential to supplement the data acquired with ICG and extend the number of candidate lymphatic vessels for LVA.

### 2.8. Tracer Design and Delivery

Contrast agents with a molecular size of 10–100 nm are considered suitable for lymphatic imaging. The currently approved tracers for human examinations, such as gadolinium-based MRI tracers, isosulphane blue, and ICG fall within this criterion, but they are located at the bottom end of the scale in terms of molecular size. Therefore, their specificity for lymphatic vessels is low [37]. (Interestingly, ICG shows better lymphatic specificity clinically due to its strong affinity for HDL, LDL, and other plasma proteins [43]). In preclinical settings, the encapsulation of the contrast agents in liposomes, micelles, calcium phosphate particles, pre-complexation with polymers, and covalently binding to larger molecules (e.g., PEG) was attempted to increase their lymphatic specificity [37].

Lymphatic contrast agents are administered interstitially (e.g., intradermal injection) or directly into the lymphatic system. There is no standardized protocol for tracer delivery in lymphology practices. One of the most common injection methods is intradermal injection into finger-webs [6]. Commercially available microneedle devices allow pain-free and controllable intradermal tracer injection (e.g., MicronJet 600 by Nanopass Ltd. (Nes Ziona, Israel) [72] and SOFUSA (Sandy Springs, GA, USA) [73]).

## 3. An Imaging-Based Lymphedema Treatment Protocol

In our tertiary care center specializing in lymphedema treatment, 376 lymphedema surgeries—360 physiological reconstructive surgeries (LVA, VLNT, and 16 debulking operations), liposuction, Charles procedure, and tissue resections—have been performed since October 2017. Considering the scientific data and the distinct characteristics of various imaging methods– shown in Table 4, we developed an algorithm for preoperative assessment.

Patients are referred from a physical therapy center, where US, mammography, CT, and MRI are used to exclude any active oncological diseases. LS, with its quantitative measurements, is carried out to confirm the diagnosis of lymphedema. The US is routinely performed to rule out venous outflow abnormalities.

Since our approach is to perform minimal invasive physiological procedures as a first step, if possible, a standardized HFUS or UHFUS examination is performed for screening candidate functional lymphatic vessels and reflux-free veins for LVA surgery in fluid-predominant regions. The LVA operation not only restores lymphatic flow but is a diagnostic procedure, where intraoperative evaluation of the lymphatic vessel state (sclerosis, ectasis, flow, and backflow) and the characteristics of surrounding tissue (fibrosis and dermis thickness) will be carried out, and if necessary, further LVA operation will be planned, based on this intraoperative diagnostic.

In total, 4–6 possible LVA spots are generally marked on the skin for each patient. Multiple lymphosome ICG-L is performed preoperatively in the operating room. We plan skin incisions based on the US and ICG mapping. In case there is an ICG uptake in the dissected lymphatic vessel, a microscope-integrated infrared fluorescence camera facilitates the dissection and ensures the patency of the anastomosis. Should we not find any lymphatic vessels with US or ICG-L for LVA surgery (1%) or LVA surgery cannot ensure a probable positive postoperative outcome, we schedule patients for VLNT operation alone or VLNT and LVA procedures combined.

## 4. Conclusions

The International Society of Lymphology (ISL) staging system for lymphedema is based just on clinical signs alone and does not provide information necessary for clinical decision-making. Objective measurements (circumference, volume, bioimpedance, dielectric constant), imaging (ultrasound—US; indocyanine green lymphography—ICG-L; lymphoscintigraphy—LS; magnetic resonance imaging—MRI; photoacoustic imaging—PAI; optical coherence tomography—OCT), and self-reporting (lymphedema life impact scale—LLFI; lower limb functional index—LEFS; disabilities of the arm, shoulder, and hand—DASH; international classification of functioning, disabilities and health—ICF; quality of life—QoL) assist in choosing the best therapy for the patient. The preoperative use of US [33], ICG-L [74], MRL [60], LS [39] solely and the combined use of US and ICG-L [27], ICGL and LS [41,75], MRL and ICG-L [55], and ICG-L and LS and MRI and CT [56] has been reported. ICG-L and MRI are superior in diagnosing lymphedema compared to LS or CT [56,57], and the US is applicable as a standalone imaging modality in the localization of LVA-candidate vessels [33]. It is suggested to use combined imaging modalities in lymphedema diagnostics and for establishing a treatment strategy [55,76]. The intraoperative use of ICG-L, PAI, or OCT can be helpful for lymphatic surgeries. Contrast-enhanced ultrasonography (CEUS), chemical exchange saturation transfer (CEST) MRI, multispectral optoacoustic tomography (MSOT), and OCT have the potential to become mainstay tools in lymphedema treatment.

## Figures and Tables

**Table 1:** Cheng’s Lymphedema Grading System helps lymphedema surgical treatment–related decision-making and is based on imaging and clinical signs [41].

Cheng’s Lymphedema Grade		Circumferential Difference (%)	Episodes of Cellulitis(Times/yr)	Taiwan Lymphoscintigraphy Staging	ICG Lymphography	Treatment
0		0–9	0–1	L-0, P-1, P-2	Patent lymphatic ducts	CDT, LVA
I	IA	10–19	1–2	P-1, P-2, P-3	Patent lymphatic ducts	LVA
	IB			P-3, T-4	Diffuse DB	VLNT
II	IIA	20–29	2–3	P-1, P-2, P-3	Patent lymphatic ducts	LVA
	IIB			P-3, T-4, T-5	Diffuse DB	VLNT
III		30–39	3–4	P-3, T-4, T-5, T-6	Not performed	VLNT + additional procedures
IV		>40	>4	T-4, T-5, T-6	Not performed	VLNT + additional procedures ^1^

CDT: complete decongestive therapy; ICG: indocyanine green lymphography; LVA: lymphovenous anastomosis; VLNT: vascularized lymph node transfer; DB: dermal backflow; ^1^ Liposuction and debulking performed in a second stage.

**Table 2:** ICG lymphography staging based on dermal backflow patterns in lower extremity [48,49].

DB Stage	ICG Lymphography Findings
Stage 0	No dermal backflow patterns
Stage I	Linear pattern + Splash pattern ^1^
Stage II	Linear pattern + Stardust pattern (1 region) ^2^
Stage III	Linear pattern + Stardust pattern (2 regions) ^2^
Stage IV	Linear pattern + Stardust pattern (3 regions) ^2^
Stage V	Stardust and/or Diffuse pattern ^3^

DB, dermal backflow; ICG, indocyanine green. ^1^ Usually seen around the groin. ^2^ Lower limb is divided into three regions: the thigh, the lower leg, and the foot. ^3^ Linear pattern is not seen.

**Table 3:** ICG lymphography staging based on dermal backflow patterns in upper extremity [52].

DB Stage	ICG Lymphography Findings
Stage 0	No dermal backflow patterns
Stage I	Splash pattern around the axilla
Stage II	Stardust pattern limited between the axilla and the olecranon
Stage III	Stardust pattern exceeding the olecranon
Stage IV	Stardust pattern observed throughout the limb
Stage V	Diffuse pattern and stardust

DB, dermal backflow; ICG, indocyanine green.

**Table 4:** Imaging modalities for lymphedema evaluation and therapy.

Modality	Applications	Advantages	Limitations
**LS**	▪ diagnosis ▪ staging ▪ surgical planning ▪ reverse lymphatic mapping	▪ (semi)quantitative and qualitative assessments ▪ deep penetration	▪ low resolution ▪ exposure to ionizing radiation ▪ high costs of producing and processing the isotope ▪ poor sensitivity ▪ large equipment ▪ contrast based
**US**	▪ exclude venous pathology ▪ surgical planning	▪ non-contrast modality (exc. CEUS) ▪ portable ▪ assess venules for LVA ▪ assess tissue composition ▪ high resolution ▪ reveals lymphatic peristalsis ▪ real-time data	▪ low penetration of UHF-US ▪ steep learning curve ▪ reproducibility ▪ time-consuming
**ICG-L**	▪ diagnosis (sensitive) ▪ staging ▪ surgical planning ▪ intraoperative tool ▪ reverse lymphatic mapping	▪ portable or microscope-integrated ▪ real-time data ▪ cost-efficient ▪ high resolution (~µm) ▪ specific for lymphatics	▪ limited detection depth ▪ specific for lymphatics (no information on the veins) ▪ suboptimal parameters of ICG dye ▪ ICG is off-label if not intravenous ▪ contrast based
**MRL**	▪ diagnosis ▪ other diagnoses (venous pathology, recurrent tumor, metastasis, lymphangiosarcoma) ▪ surgical planning	▪ visualizes lymphatics and veins ▪ no penetration-limitation ▪ accurate volumetric information ▪ information on tissue composition ▪ can be non-contrast enhanced	▪ high costs ▪ availability ▪ can be contrast based ▪ simultaneous venous enhancement (the dark-blood technique requires two contrast agents) ▪ not real-time
**PAI**	▪ staging ▪ surgical planning ▪ can be intraoperative tool	▪ can be real time ▪ multispectral (can visualize veins) ▪ microscopy: can have high resolution (low penetration) ▪ tomography: can have several cm detection depth (low resolution)	▪ contrast-enhanced (ICG) ▪ availability ▪ not-established in medicine
**OCT**	▪ intraoperative tool	▪ real-time ▪ high resolution ▪ non-contrast ▪ established in medicine	▪ low penetration ▪ not established in lymphology ▪ availability

ultrasound—US, indocyanine green lymphography—ICG-L, lymphoscintigraphy—LS, magnetic resonance imaging—MRI, photoacoustic imaging—PAI, optical coherence tomography—OCT.

## Data Availability

No new data were created or analyzed in this study. Data sharing is not applicable to this article.

## Appendix A

**Table A1:** International Society of Lymphology Stages classify lymphedema based on clinical signs.

ISL Stage	Clinical Signs or Symptoms
Stage 0	No visible swelling. The patient reports sensations associated with swelling, for example, heaviness, tightness, and aching
Stage I	Intermittent swelling that resolves overnight or with elevation. Minimal or no pitting.
Stage IIa	Visible swelling. Significant pitting. Elevation rarely resolves swelling.
Stage IIb	Visible swelling. Tissue firmness is evident in the swollen area. Minimal or no pitting.
Stage III	Large, distorted swollen area. No pitting. Very hard skin and tissue, with skin changes including extra folds, discoloration, overgrowths, and lymphorrhea.

**Table A2:** Volume difference assessment [5].

Level of Severity.	Increase in Limb Volume (%)
minimal	5–10%
mild	10–20%
moderate	20–40%
severe	40%
